# Suppression of Rat Oral Carcinogenesis by Agonists of Peroxisome Proliferator Activated Receptor γ

**DOI:** 10.1371/journal.pone.0141849

**Published:** 2015-10-30

**Authors:** David L. McCormick, Thomas L. Horn, William D. Johnson, Xinjian Peng, Ronald A. Lubet, Vernon E. Steele

**Affiliations:** 1 Life Sciences Group, IIT Research Institute, Chicago, Illinois 60616, United States of America; 2 Chemopreventive Agent Development Research Group, Division of Cancer Prevention, National Cancer Institute, Bethesda, Maryland 20852, United States of America; Winship Cancer Institute of Emory University, UNITED STATES

## Abstract

Peroxisome-proliferator-activated receptor γ (PPARγ) is a ligand-activated transcription factor that regulates cell proliferation, differentiation, and apoptosis. *In vivo* studies were performed to evaluate the activities of two thiazolidinedione PPARγ agonists, rosiglitazone and pioglitazone, as inhibitors of oral carcinogenesis in rats. Oral squamous cell carcinomas (OSCC) were induced in male F344 rats by 4-nitroquinoline-1-oxide (NQO; 20 ppm in the drinking water for 10 weeks). In each study, groups of 30 NQO-treated rats were exposed to a PPARγ agonist beginning at week 10 (one day after completion of NQO administration) or at week 17 (7 weeks post-NQO); chemopreventive agent exposure was continued until study termination at week 22 (rosiglitazone study) or week 24 (pioglitazone study). Administration of rosiglitazone (800 mg/kg diet) beginning at week 10 increased survival, reduced oral cancer incidence, and reduced oral cancer invasion score in comparison to dietary controls; however, chemopreventive activity was largely lost when rosiglitazone administration was delayed until week 17. Administration of pioglitazone (500 mg/kg diet beginning at week 10 or 1000 mg/kg diet beginning at week 17) induced significant reductions in oral cancer incidence without significant effects on OSCC invasion scores. Transcript levels of PPARγ and its three transcriptional variants (PPARγv1, PPARγv2, and PPARγv3) were not significantly different in OSCC versus age- and site-matched phenotypically normal oral tissues from rats treated with NQO. These data suggest that PPARγ provides a useful molecular target for oral cancer chemoprevention, and that overexpression of PPARγ at the transcriptional level in neoplastic lesions is not essential for chemopreventive efficacy.

## Introduction

In spite of continuing improvements in cancer therapy, oral squamous cell carcinoma (OSCC) remains a significant problem in the United States and around the world. The American Cancer Society projects that approximately 39,500 new cases of oral or oropharyngeal cancer will be diagnosed in the United States in 2015, and that approximately 7500 people will die of these cancers [1]. Approximately 30,000 of these new cases and 6000 deaths will result from cancer of the tongue, gums, lips, or floor of the mouth [1,2].

The oral cancer problem is even more significant outside of the United States, as approximately 2/3 of new oral cancer cases are diagnosed in developing countries [3]. In 2012 (the most recent year for which data are available), approximately 300,000 new cases of OSCC were diagnosed worldwide, and more than 145,000 people died of oral cancer [4]. Substantial variations in the incidence of oral cancer are seen in different parts of the world: the highest rates of OSCC occur in Melanesia, south-central Asia, and in parts of central and eastern Europe, while much lower rates are seen in western Africa and in eastern Asia [4].

Much of the variation in international rates of oral cancer appears to reflect differences in lifestyle factors that underlie disease etiology. The most important risk factors for human oral carcinogenesis are the use of tobacco and alcohol [5–9]. Recent data suggest that over 70% of OSCC diagnosed in high-income countries and nearly 40% of OSCC diagnosed in low-income and middle-income countries are related to tobacco smoking [4]. Alcohol use is identified as a causal factor in the etiology of over 30% of OSCC diagnosed in high-income countries and approximately 15% of oral cancers diagnosed in low- and middle-income countries [4]. Epidemiologic evidence suggests a synergistic interaction between tobacco and alcohol in oral cancer induction: oral cancer risk in individuals who both smoke tobacco and drink alcohol is greater than the multiplicative risk of either smoking only or drinking only [7].

Clearly, differences in smoking behavior are responsible for much of the variation in OSCC incidences seen in different countries. In addition, the use of smokeless tobacco products (chewing tobacco and snuff) is clearly linked to increased oral cancer risk [10–12], as is the use of betel quid (with or without tobacco) [12]. Both have been identified as major factors in the etiology of oral cancer in India and other central Asian countries [11,12].

Exposure to human papillomavirus (HPV) is an emerging and potentially major etiologic factor for oral cancer. Individuals infected with HPV demonstrate an increased risk of OSCC [13,14], and clinical studies demonstrate evidence of HPV infection in a significant subset of oral cancer patients [13,15]. Importantly, HPV infection has been identified as a major risk factor in the etiology of oral cancer in both younger individuals and in non-smokers and non-drinkers [14,16].

Data collected in the United States for the period of 2005 to 2011 demonstrate a 5-year survival rate of 63.2% for patients with oral or pharyngeal cancer; this compares to a 5-year survival rate of 52.7% reported in 1975 [17]. The modest improvement in 5-year survival of OSCC patients over more than four decades, when considered with morbidity and drug toxicity that are commonly associated with surgery and chemotherapy, suggest that primary prevention efforts to decrease exposure to major risk factors for OSCC (*e*.*g*., tobacco, alcohol, HPV) and secondary prevention efforts involving cancer chemoprevention are necessary to reduce mortality from this disease.

Peroxisome proliferator-activated receptor γ (PPARγ) is a ligand-activated transcription factor that plays key roles in the regulation of cell proliferation, differentiation, and apoptosis [18]. Three different PPARγ transcript variants (PPARγv1, PPARγv2, and PPARγv3) are products of the same gene, but demonstrate different promoter usage and splicing [19]. The protein product of PPARγv1 is PPARγ1; PPARγv2 and PPARγv3 code for the same protein product (PPARγ2). Although human PPARγ1 and PPARγ2 are 97% identical [19], the two PPARγ isoforms demonstrate much different tissue distributions: whereas PPARγ1 can be identified in many tissues in both humans and laboratory animals, PPARγ2 expression is limited primarily to adipocytes [20].

An expanding body of evidence supports the hypothesis that activation of signaling pathways regulated by PPARγ may provide an effective strategy to prevent and/or treat cancer (reviewed in [18,21,22]). Multiple signaling pathways and downstream cellular functions appear to be involved. Following activation by ligand binding, the formation of heterodimers between PPARγ and the retinoid “X” receptor (RXR) is a key step in its transcriptional activation [23]. Downstream effects of this activation by agonists such as thiazolidinediones include inhibition of cell proliferation, induction of apoptosis, induction of terminal differentiation, inhibition of angiogenesis, and inhibition of inflammation [18,23,24]; any (or all) of these effects could underlie chemopreventive efficacy in tissues in which PPARγ is expressed. PPARγ agonists also have important effects on insulin sensitivity and lipid metabolism [23,24]. These activities provide the mechanistic basis for the development of this class of drugs as therapeutic agents for Type II diabetes, and may also play a role in their activity in cancer chemoprevention [23].

Activation of PPARγ modulates numerous signaling pathways and associated cellular responses that are clearly linked to carcinogenesis. On this basis, PPARγ presents an attractive molecular target for cancer chemoprevention. Furthermore, the ubiquitous expression of PPARγ1 suggests that its activation by pharmacologic agents may provide a useful strategy for cancer prevention in several organ sites. To test this hypothesis, the present studies were performed to evaluate the activity of rosiglitazone and pioglitazone, two thiazolidinedione agonists of PPARγ, as inhibitors of the induction of OSCC. Studies were performed in a model for oral carcinogenesis that has been used in chemoprevention efficacy evaluations of numerous other classes of agents (reviewed in [25]).

## Materials and Methods

Prior to the initiation of *in vivo* studies, study protocols were reviewed and approved by the IIT Research Institute Animal Care and Use Committee. All aspects of the program involving animal care, use, and welfare were performed in compliance with United States Public Health Service Policy on Humane Care and Use of Laboratory Animals, and in compliance with the guidelines stated in the National Research Council *Guide for the Care and Use of Laboratory Animals*. During twice daily mortality/moribundity checks, sixteen criteria for animal euthanasia are used to identify animals that should be considered for euthanasia. These criteria are consistent with the American Veterinary Medical Association Guidelines for the Euthanasia of Animals (2013 Edition). Specific criteria for euthanasia that were used in the present studies included: loss of > 15% of body weight in one week; gradual but continuous decline in body weight; adequate indication that a study animal may not survive until the next scheduled observation; and prolonged unhealthy appearance such as rough coat or hunched posture.

Separate *in vivo* carcinogenesis studies were performed to evaluate the chemopreventive efficacy of rosiglitazone and pioglitazone. The dose levels of each agent used in chemoprevention studies were selected on the basis of the results of a preliminary toxicity/dose selection study of that agent. The goals of dose selection studies were to identify dietary levels of each agent that induced no mortality, body weight suppression, or other evidence of limiting toxicity.

### Animal receipt, housing, and quarantine

Male F344 rats were received at approximately seven weeks of age from virus-free barrier colonies either: (a) maintained under contract to the National Cancer Institute, Frederick, MD (rosiglitazone studies); or (b) at Charles River Laboratories, Raleigh NC (pioglitazone studies). After receipt from the vendor, rats were held in quarantine for a minimum of one week prior to randomization into a dose selection or oral cancer chemoprevention study. Prior to release from quarantine and randomization into a study, each rat underwent a hand-held clinical and physical examination to ensure its suitability for use as a test subject.

Rats were housed in pairs or trios on hardwood bedding in polycarbonate shoebox cages in a windowless room that was illuminated for 12 hours each day and maintained at 22 ± 1°C and within the range of 30% to 70% relative humidity. Throughout all studies, rats were permitted free access to Purina 5001 Laboratory Diet (PMI Feeds, Brentwood, MO) and City of Chicago drinking water (provided in water bottles).

### Dose Selection Studies

A preliminary toxicity/dose selection study was performed for each agent to select dose levels for use in the chemoprevention study. In each dose selection study, groups of 10 male F344 rats received diets supplemented with rosiglitazone or pioglitazone at doses of 0, 200, 400, 600, 800, or 1000 mg/kg diet for 6 weeks. Rats were observed twice daily to identify any gross clinical evidence of toxicity; body weight was measured individually for each rat once per week. At the end of the exposure period, each rat was humanely euthanized with CO_2_ and subjected to a limited gross necropsy to identify any gross pathology associated with agent exposure.

### Chemoprevention studies

Hyperplastic and neoplastic lesions were induced on the tongue of male F344 rats by drinking water administration of 4-nitroquinoline-1-oxide (NQO), as described by Tanaka (reviewed in [25]) and used in previous oral cancer prevention studies performed in our laboratory [26]. NQO (Sigma-Aldrich, St. Louis, MO), was stored in the dark at -20°C until used. Drinking water formulations of NQO (20 ppm) were prepared every two weeks, and were stored in the dark at 4°C until used. Bottles containing NQO-supplemented drinking water were wrapped with foil to preclude possible photodegradation of the carcinogen. Animals received drinking water exposure to NQO for 10 weeks; bottles containing NQO-supplemented water were changed at two- to three-day intervals throughout the administration period.

After release from quarantine, rats were assorted into groups of 30 using a constrained randomization procedure that blocks for body weight. Rosiglitazone and pioglitazone were obtained from the Chemopreventive Agent Repository maintained by the Division of Cancer Prevention, National Cancer Institute. Dietary administration of each chemopreventive agent was initiated either: (a) one day after completion of NQO exposure (study week 10); or (b) 7 weeks after completion of NQO exposure (study week 17). Once initiated, administration of chemopreventive agents was continued until study termination at week 22 (rosiglitazone study) or week 24 (pioglitazone study).

Cage-side observations were performed a minimum of twice daily to evaluate animal health and identify possible toxicities resulting from administration of NQO or PPARγ agonists. Rats were weighed weekly throughout the studies. Body weight is a key metric in oral cancer chemoprevention studies in the NQO model, since body weight loss provides a useful indication of the clinical progression of OSCC [25]. Hand-held observations were performed weekly to monitor the presence of oral lesions.

At the termination of each chemoprevention study; surviving rats were humanely euthanized by CO_2_ asphyxiation. All study animals (whether found dead, euthanized *in extremis*, or euthanized at study termination) received a limited gross necropsy focused on the tongue and oral cavity. The tongue from each rat was carefully excised, all gross lesions were charted, and the tongue was bisected longitudinally. At necropsy, half of each tongue was fixed in 10% neutral buffered formalin for histopathologic evaluation, and the other half of each tongue was snap-frozen in liquid nitrogen and stored at -80°C for use in molecular analyses. To avoid possible artifacts due to post-mortem changes, only tissues from freshly euthanized animals were used for molecular studies.

Each bisected tongue was processed by routine histologic methods, and replicate sections were cut at 5 μm and stained with hematoxylin and eosin. The histopathologic classification of each lesion and its depth of invasion were determined in order to (a) quantify tumor incidence and (b) determine if a shift to a less invasive lesion may have occurred as a result of chemopreventive agent administration. Cancer invasion was classified using a semi-quantitative grading system [27]. In this system, non-invasive lesions were assigned a score of 0; lesions extending through the epithelial basement membrane and into the lamina propria only were assigned an invasion score of +1; lesions extending through the lamina propria into upper muscle layers were assigned an invasion score of +2; and the most invasive lesions, which demonstrated extensive infiltration into underlying muscle, were assigned an invasion score of +3. Photomicrographs of oral lesions induced by NQO have recently been published [27].

### Analysis of PPARγ expression

#### Microarray Analysis

Microarray analysis was performed on 11 pairs of histologically confirmed oral cancers and adjacent phenotypically normal oral tissues from dietary control rats treated with NQO. After tissues were removed from storage at -80°C, RNA was isolated, and the quantity and quality of each RNA sample was determined using an Agilent Bioanalyzer (Agilent Technologies, Santa Clara, CA). Gene expression in each tissue pair was then analyzed using Agilent Rat GE 4x44K v3 arrays (Agilent Technologies, Santa Clara, CA). First and second strand cDNAs were prepared, cRNA target was prepared from the DNA template, verified, fragmented to uniform size, and hybridized to the microarrays. Slides were washed and scanned using an Agilent G2565 Microarray Scanner. Microarray data were analyzed using Agilent Feature Extraction and GeneSpring GX v7.3.1 software packages. Microarray data have been deposited in the National Center for Biotechnology Information Gene Expression Omnibus (GEO accession GSE51125).

#### PCR Analysis

RT-PCR analyses were performed to compare the relative abundance of total PPARγ and its variants (PPARγv1, PPARγv2, and PPARγv3) in fifteen pairs of age- and site matched normal and neoplastic oral tissues. Tissues used for RT-PCR analyses were harvested from NQO-treated dietary control rats from a different study than tissues used for microarray analysis. Total RNA was isolated from these tissues by homogenization in 0.2 ml TRIzol reagent (Life Technologies, Carlsbad, CA), followed by extraction using an additional 0.8 ml TRIzol. RNA integrity was determined by 1% agarose gel electrophoresis; purity was assessed by spectrophotometry.

Gene-specific primers were designed using Primer 3 software (http://bioinfo.ut.ee/primer3-0.4.0/); primer sequences are provided in Table 1. 200 ng total RNA from each sample was used for reverse transcription (RT) reactions in 20 μl reaction volume. Two RT reactions were pooled and diluted by 4 fold using DNase/RNase-free water. Real-time PCR was performed with 2 μL diluted RT products (cDNA) in a CFX96 Real-Time PCR Detection System (Bio-Rad, Hercules, CA) using iQ SYBR Green PCR Supermix (Bio-Rad). Cq values for each sample were determined using CFX Manager 3.0 software (Bio-Rad). Relative gene expression was calculated using the formula 2^-(∆Cq)^, where ∆Cq is Cq(sample)—Cq(HSP90ab1). HSP90ab1 was used as a common reference gene for normalization; we have recently identified HSP90ab1 as a reliable reference gene in this model. To ensure the specificity of PCR for each primer pair, melting curve analysis was performed after PCR reactions.

**Table 1:** Primer Sequences used in PCR Analyses of PPARγ Expression.

Gene	Forward Primer Sequence	Reverse Primer Sequence
PPARγ (total)	TTCAGAAGTGCCTTGCTGTG	CCAACAGCTTCTCCTTCTCG
PPARγv1	GTGCCTTCGCTGATGCACTG	CAGAGAGGTCCACAGAGCTGA
PPARγv2	AAGGCTGCAGCGCTAAATTC	ATGGCATCTCTGTGTCAACCAT
PPARγv3	CTTTCTGACCGGACTGTGTG	ATGGCATCTCTGTGTCAACCAT
HSP90ab1 (reference gene)	CACCCTGCTCTGTACTACTACTC	GGGCAATTTCTGCCTGAAAGG

### Statistical analysis

Evidence of chemopreventive activity was defined as a statistically significant (*p* < 0.05) reduction in oral cancer incidence, reduction in oral cancer invasion score, or increase in survival in a carcinogen-treated group receiving a PPARγ agonist when compared to the carcinogen-treated dietary control group. Comparisons of OSCC incidence were performed using X^2^ analysis. Because oral cancer invasiveness was evaluated using a semi-quantitative scoring system, comparisons of invasion scores were performed using non-parametric statistics (Wilcoxon rank-sum analysis). Animal survival was evaluated using X^2^ analysis. Body weights and other continuous data were compared by analysis of variance, with *post-hoc* comparisons made using Dunnett's test.

## Results

### Dose Selection Studies of Rosiglitazone and Pioglitazone

Dietary administration of rosiglitazone at levels of up to 1000 mg/kg diet for six weeks induced no evidence of toxicity in male F344 rats. No mortality was observed during the rosiglitazone dose selection study, and no clinical signs of toxicity or gross pathology were identified in any study animal. Rosiglitazone did induce a dose-related and statistically significant increase in mean body weight in all groups: in comparison to dietary controls, statistically significant increases in group mean body weight were first seen at week 2 in groups fed rosiglitazone at doses ≥ 600 mg/kg diet, at week 5 in the group fed rosiglitazone at 400 mg/kg diet, and at week 6 in the group fed rosiglitazone at 200 mg/kg diet.

Similarly, dietary administration of pioglitazone at levels of up to 1000 mg/kg diet for six weeks induced no evidence of toxicity in male F344 rats. No mortality was seen in any group in the study, and no clinical signs of toxicity or gross pathology at the terminal necropsy were observed. Although mean terminal body weights were increased from control in all groups exposed to pioglitazone, the differences were not statistically significant at any point in the study.

### Chemoprevention Efficacy Evaluation of Rosiglitazone

In the chemoprevention efficacy evaluation of rosiglitazone, groups of 30 NQO-treated F344 rats received either unsupplemented basal diet (control), diet supplemented with rosiglitazone (800 mg/kg diet) from weeks 10 to 22 (normal intervention schedule), or diet supplemented with rosiglitazone (800 mg/kg diet) during weeks 17 to 22 (delayed intervention schedule). The 800 mg/kg diet dose level of rosiglitazone was selected on the basis of the results of a preliminary toxicity/dose selection study.

Administration of rosiglitazone beginning at week 10 conferred significant protection against the induction of OSCC by NQO (Table 2). Chemopreventive activity was demonstrated as a statistically significant decrease in oral cancer incidence and a statistically significant increase in animal survival.

**Table 2:** Influence of Rosiglitazone on Survival and Incidence of Preneoplastic and Neoplastic Oral Lesions in NQO-Treated F344 Rats.

Group	Rosiglitazone Dose (mg/kg diet)	Exposure Schedule (Weeks)	Survival^a^ (%)	Lesion Number (% Incidence)			
				Normal	Squamous Epithelial Hyperplasia	Squamous Cell Papilloma	Squamous Cell Carcinoma
1	0 (Control)	—	22/30 (73)	1/29 (3)	2/29 (7)	0/29 (0)	26/29 (90)
2	800	10–22	27/30 (90)**	3/30 (10)	4/30 (13)	4/30 (13)**	19/30 (63)**
3	800 (late)	17–22	25/30 (83)	1/30 (3)	4/30 (13)	0/30 (0)	25/30 (83)

^a^ all early mortality was the result of oral neoplasia. 74/90 rats survived until the terminal necropsy, 9 rats were euthanized *in extremis*, and 7 rats were found dead prior to the terminal necropsy

** p < 0.05 versus control

In comparison to a final oral cancer incidence of 90% (26/29 rats) in the dietary control group, the incidence of OSCC in rats receiving rosiglitazone from weeks 10 to 22 was 63% (19/30; *p* < 0.05). The decrease in the incidence of OSCC in rats receiving rosiglitazone by the normal intervention schedule (weeks 10 to 22) was accompanied by a statistically significant increase in the incidence of squamous cell papillomas, a putative preneoplastic lesion, and a non- significant increase in the incidence of squamous epithelial hyperplasia (Table 2).

Previous data from studies conducted in our laboratory have shown that essentially all mortality in the NQO OSCC model system is associated with oral cancer development; this result was confirmed in the oral cancer chemoprevention study with rosiglitazone. In addition to reducing OSCC incidence, dietary administration of rosiglitazone beginning at week 10 resulted in a statistically significant increase in survival (90% (27/30) versus 73% (22/30) in the dietary control group; p < 0.05).

Administration of rosiglitazone beginning at week 10 also induced a statistically significant shift in OSCC invasion scores (Table 3). Whereas 4% (1/26) of cancer-bearing rats in the dietary control group demonstrated OSCC with the lowest (+1) invasion score, 32% (6/19) of cancer-bearing rats receiving rosiglitazone from weeks 10 to 22 demonstrated OSCC with +1 invasion scores (p < 0.05). Conversely, 69% (18/26) of cancer-bearing rats in the vehicle control group demonstrated OSCC with the highest (+3) invasion score. By contrast, +3 invasion scores were seen in only 9/19 (47%) of cancer-bearing rats in the group receiving rosiglitazone beginning at week 10.

**Table 3:** Influence of Rosiglitazone on Oral Cancer Invasion Scores in NQO-Treated F344 Rats.

Group	Rosiglitazone Dose (mg/kg diet)	Exposure Schedule (Weeks)	Lesion Number (% Incidence)		
			+1 Invasion	+2 Invasion	+3 Invasion
1	0 (Control)	—	1/26 (4)	7/26 (27)	18/26 (69)
2	800	10–22	6/19 (32)**	4/19 (21)	9/19 (47)
3	800 (late)	17–22	5/25 (20)*	7/25 (28)	13/25 (52)

* 0.05 < p < 0.10 versus control

** p < 0.05 versus control

Although administration of rosiglitazone beginning at study week 10 conferred significant protection against oral carcinogenesis, delaying the start of rosiglitazone exposure until week 17 resulted in the loss of much of its chemopreventive activity. When compared to dietary controls, the group receiving delayed administration of rosiglitazone demonstrated a small reduction in oral cancer incidence (Table 2) and a modest reduction in cancer invasion scores (Table 3); neither of these differences was significantly different from dietary control. Similarly, the increased survival seen in the group receiving rosiglitazone beginning at week 17 (late intervention) did not achieve statistical significance (Table 2).

**Fig 1:**
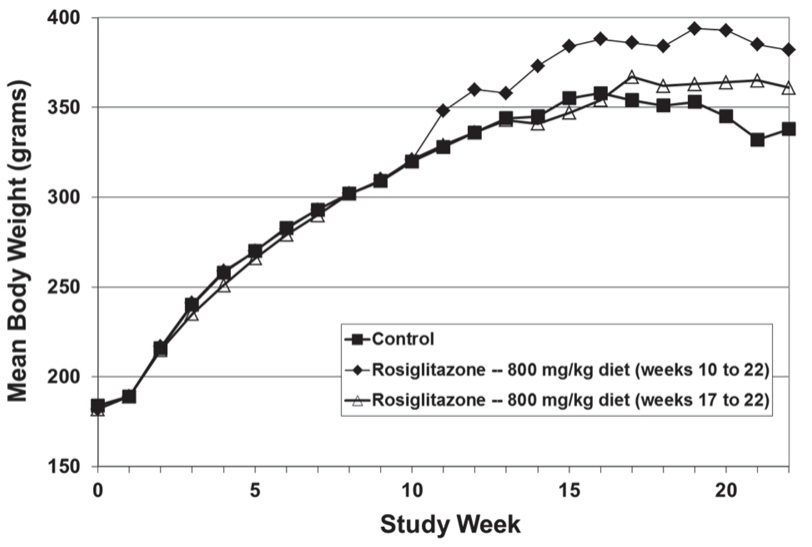
Influence of Rosiglitazone on Group Mean Body Weight in NQO-Treated Rats. Rosiglitazone (800 mg/kg diet) was administered to NQO-treated rats beginning either at week 10 (1 day after completion of NQO administration) or at week 17 (7 weeks after completion of NQO administration; late administration group). In comparison to the NQO-treated control group, statistically significant increases in group mean body weight were seen in rosiglitazone-treated rats at the following times: 800 mg rosiglitazone per kg diet group, weeks 11, 12, and 14 through 22; 800 mg rosiglitazone per kg diet (late) group, week 21 only.

Changes in body weight provide a useful marker for oral cancer incidence and chemopreventive efficacy in this model. Actively growing NQO-induced oral cancers are commonly associated with body weight loss during later weeks of chemoprevention studies [26]; these changes may result from interference with normal feeding patterns due to the strategic location of the tumor, effects of the tumor on host metabolism, and/or from tumor-associated cachexia. Administration of rosiglitazone beginning immediately after the cessation of carcinogen exposure (week 10) increased group mean body weight throughout the study, and prevented the tumor-associated loss of body weight seen late in the experiment in dietary controls (*p* < 0.05; Fig 1).

Administration of rosiglitazone beginning at week 17 had no statistically significant effect on group mean body weight, but did prevent the apparent tumor-associated body weight loss seen in dietary controls between weeks 19 and 22 of the study (Fig 1).

### Chemoprevention Efficacy Evaluation of Pioglitazone

In the chemoprevention efficacy evaluation of pioglitazone, groups of 30 NQO-treated rats received either unsupplemented basal diet (control); diet supplemented with the high dose of pioglitazone (1000 mg/kg diet) from weeks 10 to 24 (normal intervention schedule); diet supplemented with the low dose of pioglitazone (500 mg/kg diet) from weeks 10 to 24; or diet supplemented with the high dose of pioglitazone from weeks 17 to 24 (delayed intervention schedule). The high dose of pioglitazone (1000 mg/kg diet) was selected on the basis of the results of a preliminary toxicity/dose selection study.

Statistically significant reductions in the incidence of OSCC were seen in groups receiving dietary administration of the low dose (500 mg/kg diet) of pioglitazone and delayed administration of the high dose (1000 mg/kg diet) of pioglitazone (Table 4; *p* < 0.05 for both comparisons). In both groups, the lower incidence of invasive oral squamous cell carcinomas was accompanied by an apparently compensatory increase in the incidence of squamous epithelial hyperplasia, a putative precursor lesion in this experimental model (0.05 < p < 0.10 for both comparisons). Unexpectedly, when administered by the standard intervention protocol, the high dose of pioglitazone had no significant effect on oral carcinogenesis, and also did not increase the incidence of oral squamous epithelial hyperplasia. When compared to dietary controls, pioglitazone had no statistically significant effect on oral cancer invasion scores in any group (Table 5).

**Table 4:** Influence of Pioglitazone on Survival and Incidence of Preneoplastic and Neoplastic Oral Lesions in NQO-Treated F344 Rats.

Group	Pioglitazone Dose (mg/kg diet)	Exposure Schedule (Weeks)	Survival ^a^ (%)	Lesion Number (% Incidence)			
				Normal	Squamous Epithelial Hyperplasia	Squamous Cell Papilloma	Squamous Cell Carcinoma
1	0 (Control)	—	20/30 (67)	0/30 (0)	2/30 (7)	1/30 (3)	27/30 (90)
2	500	10–23	25/30 (83)	0/30 (0)	7/30 (23)*	3/30 (10)	20/30 (67)**
3	1000	10–23	20/30 (67)	1/30 (3)	3/30 (10)	2/30 (7)	24/30 (80)
4	1000 (late)	17–23	15/30 (50)	0/29 (0)	7/29 (24)*	2/29 (7)	20/29 (69)**

^a^ all early mortality was the result of oral neoplasia. 80/120 rats survived until the terminal necropsy, 11 rats were euthanized *in extremis*, and 29 rats were found dead prior to the terminal necropsy

* 0.05 < p < 0.10 versus control

** p < 0.05 versus control

**Table 5:** Influence of Pioglitazone on Oral Cancer Invasion Score in NQO-treated F344 Rats.

Group	Pioglitazone Dose (mg/kg diet)	Exposure Schedule (Weeks)	Lesion Number (% Incidence)		
			+1 Invasion	+2 Invasion	+3 Invasion
1	0 (Control)	—	1/30 (3)	3/30 (10)	22/30 (73)
2	1000	10–23	2/30 (7)	3/30 (10)	19/30 (63)
3	500	10–23	2/30 (7)	3/30 (10)	15/30 (50)
4	1000 (late)	17–23	0/29 (0)	2/29 (7)	18/29 (62)

**Fig 2:**
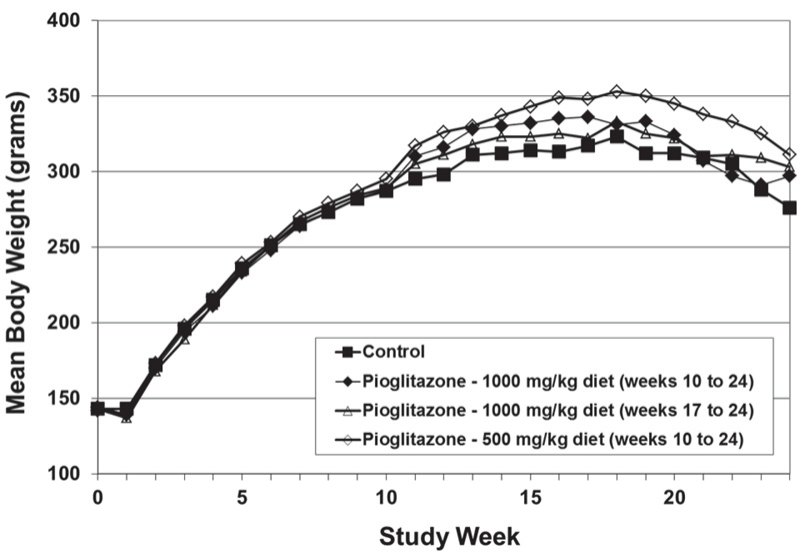
Influence of Pioglitazone on Group Mean Body Weight in NQO-Treated Rats. Pioglitazone was administered at 500 or 1000 mg/kg diet to NQO-treated rats beginning at week 10 (1 day after completion of NQO administration), or at 1000 mg/kg diet beginning at week 17 (7 weeks after completion of NQO administration; late administration group). In comparison to the NQO-treated control group, statistically significant increases in group mean body weight were seen in pioglitazone-treated rats at the following times: 500 mg pioglitazone per kg diet, weeks 12 through 21; 1000 mg pioglitazone per kg diet, week 12, 14, 15, and 16; 100 mg pioglitazone per kg diet (late), no statistically significant differences at any time point.

Body weights in all groups exposed to pioglitazone were greater than body weights in dietary controls throughout the study (Fig 2); however differences in group mean body weights reached statistical significance only in the low dose pioglitazone group from weeks 12 through 21 and in the high dose pioglitazone group between weeks 12 and 16. Because the effects of pioglitazone on body weight did not demonstrate clear evidence of a dose-response relationship, they could not be clearly related to agent exposure. Importantly, because body weights in all pioglitazone-treated groups were greater than those in dietary controls, no direct evidence of pioglitazone toxicity can be inferred.

A similar lack of dose-relatedness was seen in animal survival (Table 4). At study termination, survival in the high dose pioglitazone group was identical to that in vehicle controls (20/30 rats). By contrast, survival in the low dose pioglitazone group was increased (25/30) versus controls, while survival in the high dose delayed intervention group (15/30) was reduced from control levels. None of these differences in survival was statistically significant (*p* > 0.10 for all comparisons).

### Comparative Expression of PPARγ in OSCC versus Normal Oral Tissues

Microarray analyses of 11 pairs of OSCC and histologically normal oral tissues collected from NQO-treated rats failed to demonstrate any differential expression of PPARγ. In these tissue pairs, the mean level of PPARγ transcripts in OSCC was 101.4 ± 4.4% of the mean level of PPARγ transcripts in phenotypically normal oral tissues (p = 0.395; Table 6).

**Table 6:** Comparative Expression of PPARγ and PPARγ Transcript Variants in OSCC and Normal Oral Tissues from NQO-Treated Rats.

Gene Symbol	Method of Analysis	Relative fold change (OSCC/normal)	p value
PPARγ	Microarray	1.01 ± 0.44	0.395
PPARγ	RT-PCR	1.21 ± 0.59	0.309
PPARγv1	RT-PCR	2.63 ± 3.61	0.745
PPARγv2	RT-PCR	1.48 ± 0.91	0.132
PPARγv3	RT-PCR	0.94 ± 0.33	0.524

Although microarray data did not demonstrate significant differential expression of PPARγ in rat oral cancers, KEGG analysis of microarray data from these samples did identify statistically significant differential expression of PPARγ-associated signaling pathways in OSCC. Analysis of microarray data using Genespring software identified 19 PPARγ-associated genes whose expression was significantly different in OSCC versus paired histologically normal oral tissues (p = 3.89 x 10^−5^). Most of the genes that were identified as differentially expressed in OSCC are involved in lipid metabolism: significantly down-regulated genes included CD36 (thrombospondin receptor); acyl-CoA dehydrogenases (C-4 to C-12 straight chain and long chain); acyl-coA synthetases (long chain family members 1 and 6); acyl co-A oxidases (branched chain); and carnitine palmitoyltransferases 1b and 2. Significantly up-regulated genes in OSCC included angiopoietin-like 4, adiponectin, and fatty acid desaturase 2. The roles, if any, of these genes in the etiology and biology of OSCC remain to be defined.

PPARγ expression data from microarray analyses were confirmed and extended by PCR analysis of transcript levels in 15 different sets of OSCC and paired normal oral tissues. Mean expression of total PPARγ in this set of OSCC was 121 ± 59% of that seen in paired phenotypically normal oral tissues from NQO-treated rats (Table 6). Similarly, no statistically significant differential expression of PPARγv1, PPARγv2, or PPARγv3 was seen in comparisons of OSCC versus normal oral tissues (Table 6). In this regard, it should be noted that although PCR data identified a mean 2.6-fold up-regulation of PPARγv1 expression in OSCC, expression of PPARγv1 in normal oral tissues is very low and expression of PPARγv1 in OSCC was found to be highly variable. For this reason, the 2.6-fold increase in levels of PPARγv1 transcripts in OSCC was not significant at the 5% level of confidence. Similarly, neither PPARγv2 nor PPARγv3 was differentially expressed in rat OSCC induced by NQO. PPARγv3 was the most abundant PPARγ variant dentified in phenotypically normal oral tissues and OSCC.

## Discussion

The oral cavity has several attributes that make it an attractive site for clinical efforts in cancer prevention. Key risk factors for OSCC are well-known, and can be used to identify high risk individuals who are most likely to benefit from a chemopreventive intervention. The accessibility of the oral cavity permits either local or systemic administration of chemopreventive agents, and also allows the facile identification and monitoring of preneoplastic lesions (leukoplakia and erythroplakia) that can be used to characterize disease progression and agent efficacy. Convenient access to preneoplastic and neoplastic oral lesions also facilitates collection of tissues for histopathology, biochemical testing, or molecular analyses.

The present studies were performed to evaluate the hypothesis that neoplastic development in the oral cavity can be inhibited or delayed by pharmacologic activation of PPARγ action. To address this hypothesis, *in vivo* studies were performed to determine the chemopreventive activity of rosiglitazone and pioglitazone, two thiazolidinedione compounds that are well-studied agonists of PPARγ. We report that rosiglitazone and pioglitazone both confer statistically significant protection against carcinogenesis in a well-studied rat model for OSCC. Chemopreventive efficacy was expressed as a statistically significant reduction in oral cancer incidence (rosiglitazone and pioglitazone); a statistically significant reduction in oral cancer invasiveness (rosiglitazone); and a statistically significant reduction in oral cancer-related mortality (rosiglitazone). Although a substantial number of PPARγ-associated molecular pathways were differentially expressed in OSCC in comparison to site-matched phenotypically normal oral tissues, neither total PPARγ nor any PPARγ transcript variant was significantly overexpressed at the RNA level in OSCC. On this basis, it is concluded that the chemopreventive efficacy of the two PPARγ agonists evaluated in these studies is not dependent on overexpression of PPARγ or any PPARγ transcript variant in OSCC.

Although rosiglitazone and pioglitazone both demonstrate significant activity as chemopreventive agents for oral cancer in the NQO/F344 rat model, the toxicity of these agents in humans makes them unacceptable for possible clinical use in cancer prevention. Clinical administration of thiazolidinediones has been linked to several serious toxicities in humans [28], including increased risks of myocardial infarction and heart failure [29]. In addition, a Working Group convened by the International Agency for Research on Cancer recently classified pioglitazone as a Group 2A carcinogen (probably carcinogenic to humans) on the basis of what was deemed to be sufficient evidence of pioglitazone carcinogenicity in laboratory animals and a positive association between the use of pioglitazone and cancer of the urinary bladder in humans [30]. The same Working Group concluded that rosiglitazone could not be classified as to its human carcinogenicity (Group 3), based largely on the lack of adequate evidence of carcinogenicity in clinical studies [30]. However, the Working Group did conclude that limited evidence of rosiglitazone carcinogenicity in laboratory animals has been reported. This conclusion was based on treatment-related increases in the incidences of subcutaneous lipoma in male and female rats in a two-year carcinogenicity study [31], and the apparent enhancement of urinary bladder carcinogenesis in rats previously exposed to the chemical carcinogen, *N*-butyl-*N*-(4-hydroxybutyl)nitrosamine [32].

Although rosiglitazone and pioglitazone are not appropriate candidates for use in human cancer chemoprevention, it has been suggested that the clinical toxicities reported for these and other PPARγ modulators may be compound-specific rather than the result of the mechanism of action of the drug class [28]. For this reason, and in consideration of the significant efficacy of both rosiglitazone and pioglitazone as inhibitors of oral carcinogenesis, PPARγ remains a potentially valuable molecular target for cancer chemoprevention in the oral cavity and other sites [22,28]. To this end, we propose that novel PPARγ agonists that are suitable for cancer chemoprevention could be developed using strategies that include: (a) synthesis of new PPARγ agonists based on platforms such as *N*-acetylfarnesylcysteine [33] or isoquinolines such as berberine [34] that are structurally distinct from the thiazolidinediones; (b) development of agents with dual agonist activity (*e*.*g*., dual PPARα/γ agonists such the glitizars [27,35]); and/or (c) development of partial agonists of PPARγ (*e*.*g*., *N*-(4-hydroxyphenethyl)-3-mercapto-2-methylpropanamide [36]) that retain desired downstream effects of PPARγ agonist activity while demonstrating reduced toxicity. A different strategy towards the same goal would be to develop small molecules that can modulate PPARγ expression via manipulation of its phosphorylation status [37].

The chemopreventive activity of PPARγ agonists in the oral cavity and other epithelial tissues may be mediated by effects on a substantial number of signaling pathways, including several that play key regulatory roles in inflammation, cell proliferation, and carcinogenesis. Treatment with a PPARγ agonist has been reported to reduce COX-2 expression in human prostate cancer cells *in vitro* [38]; we have previously reported that both a specific COX-2 inhibitor (celecoxib) and several non-specific COX inhibitors (piroxicam, naproxen, NO-naproxen) are potent chemopreventive agents in the NQO/F344 rat oral carcinogenesis model [26]. Similarly, a mixture of dietary tocopherols that increases PPARγ expression has been shown to decrease levels of inflammatory markers in the serum of estrogen-treated ACI rats [39]. The chemopreventive activity of PPARγ agonists may also involve effects on angiogenesis; rosiglitazone and pioglitazone have been reported to inhibit VEGF- and bFGF-induced angiogenesis *in vitro* [40]. It should also be noted that a substantial number of natural products with demonstrated chemopreventive activity in animal models have been shown to activate PPARγ; these natural products include resveratrol, green tea catechins, tocotrienols, quercetin, genistein, and isoflavones [37]. Clearly, the possible role of PPARγ in carcinogenesis and as a target for chemopreventive drug development extends well-beyond its documented effects on host metabolism.

The results of the present studies suggest that additional agonists or partial agonists of PPARγ merit consideration for efficacy evaluation in preclinical models of oral carcinogenesis. In consideration of the clinical toxicity of rosiglitazone and pioglitazone, emphasis should be placed on natural products and new molecular entities whose structures are unrelated to thiazolidinediones. Should a novel agent or natural product demonstrate both significant chemopreventive activity in preclinical models and a toxicity profile that is consistent with use in healthy or at-risk individuals (rather than patients with a diagnosis of oral cancer), clinical studies to evaluate the efficacy of that agent in the prevention of human oral cancer could provide a valuable addition to clinical strategies to control this malignancy.
